# Polymyalgia rheumatica following COVID-19 vaccination: Case series of 3 patients and literature review on polymyalgia rheumatica induced by various vaccines

**DOI:** 10.1097/MD.0000000000040204

**Published:** 2024-10-25

**Authors:** Laure Irani, Mira Bou Karroum, Yara Chehab, Nesrine Abi Saad, Ali Al Dailaty, Roula Husni

**Affiliations:** a Division of Rheumatology, Department of Internal Medicine, LAU Medical Center – Rizk Hospital, Zahar St., Achrafieh, Beirut, Lebanon.

**Keywords:** Ad26.COV2.S, BNT162b2, ChAdOx1-S, COVID-19, immune-mediated, inflammatory disorder, mRNA-1273, polymyalgia rheumatica

## Abstract

**Rationale:**

Since the onset of the severe acute respiratory syndrome coronavirus-2 (SARS-CoV-2) pandemic in 2019, considerable resources have been devoted to developing vaccines to reduce related deaths and the burden of disease. Various vaccine formulations eventually became available and were approved for clinical use. In this article, we have conducted a review of polymyalgia rheumatica (PMR) cases induced by different COVID-19 vaccines [Pfizer: BNT162b2, AstraZeneca: ChAdOx1-S, Moderna: mRNA-1273, and Janssen: Ad26.COV2.S)], as well as non-COVID-19 vaccines, such as influenza, zoster, hepatitis B, and tetanus vaccines. Additionally, this article investigates 3 cases with clinical presentations suggestive of PMR following COVID-19 mRNA vaccination. This study aims to offer valuable insights through sharing diagnostic and therapeutic experiences.

**Patient concerns:**

Three patients presented with severe pain and stiffness in both shoulder and pelvic girdle muscles, following COVID-19 mRNA vaccination.

**Diagnoses:**

Clinical presentations, laboratory parameters, and echographic findings confirmed the diagnosis of PMR following COVID-19 mRNA vaccination.

**Interventions:**

Patients received Prednisone and/or Methotrexate adjusted to body weight.

**Outcomes:**

Polymyalgia rheumatica resolved successfully without any adverse events.

**Lessons:**

Although direct causality was not definitively established in this article, the BNT162b2 COVID-19 mRNA vaccine, similar to other vaccines, might be considered a potential trigger for PMR. This raises the need for further research into this issue and potentially other immunological outcomes.

## 1. Introduction

Polymyalgia rheumatica (PMR) is an inflammatory disorder with an etiology that is still debated. It typically affects individuals over 50 years of age, causing stiffness and pain in the shoulder and pelvic girdle muscles. Activation of both the innate and adaptive immune responses following exposure to environmental triggers is believed to contribute to the disease’s pathogenesis.^[1–9]^ Commonly reported triggers include viral infections, such as SARS-CoV-2 and other viruses, as well as vaccines.^[1–9]^

In this report, we present 3 cases of polymyalgia rheumatica following the administration of the BNT162b2 coronavirus disease 2019 (COVID-19) messenger ribonucleic acid (mRNA) vaccine. Additionally, we review the literature on PMR induced by other COVID-19 vaccines: ChAdOx1-S, mRNA-1273, and Ad26.COV2.S, as well as by non-COVID vaccines: influenza, hepatitis B, zoster, and tetanus.

## 2. Case presentations

### 2.1. Case 1

A 55-year-old Caucasian male with a history of hypertension, prediabetes, obesity with a body weight of 106 kg, obstructive sleep apnea, kidney stones, and gout, visited the outpatient clinic of LAUMCRH hospital in July 2021, 2 weeks after receiving his second dose of the BNT162b2 COVID-19 mRNA vaccine. The chief complaint was severe pain in both shoulder and pelvic girdle muscles. Associated symptoms included a low-grade fever, generalized fatigue, and nighttime sleep disturbances due to pain. Additionally, he experienced morning stiffness that lasted more than 60 minutes and improved with physical activity throughout the day. Upon presentation, multiple COVID-19 polymerase chain reaction tests were conducted; however, all yielded negative results. The patient reported difficulty dressing himself without help in the morning and challenges in getting up from a seated position after long drives. No signs of giant cell arteritis (GCA) were evident at the time. Family history includes a younger brother with ankylosing spondylitis. The physical exam revealed a decreased active range of motion in the shoulders and pelvic girdles, with tenderness upon palpation of the proximal arms and hip muscles. Laboratory tests conducted in July 2021 showed increased inflammatory markers: Erythrocyte sedimentation rate (ESR): 40 mm/h, C-reactive protein (CRP): 4.2 mg/dL (normal < 0.5 mg/dL). The complete blood count with differential was normal. Rheumatoid factor (RF) and anti-citrullinated protein antibody (ACPA) were both negative. Clinical and laboratory diagnosis of PMR was confirmed, and the patient was started on prednisone 20 mg once daily for 1 month, with the dosage adjusted according to his body weight. On a follow up visit 1 month later, the patient reported a rapid improvement in his symptoms. Laboratory tests showed a decrease in ESR to 15 mm/h and a decrease in CRP to 1.03 mg/dL. Two weeks later, a follow up showed nearly 100% improvement in symptoms and normal inflammatory markers. After 5 months of tapering his dose, the patient was still receiving 7.5 mg of prednisone daily and continues to do well.

### 2.2. Case 2

A 55-year-old Caucasian woman with a history of hypothyroidism, migraines over the past year, and fatty liver disease visited the outpatient clinic at LAUMCRH hospital due to sudden onset of pain in her elbows, shoulders, hips, and knees. This started 1 day after she received her second dose of the BNT162b2 COVID-19 mRNA vaccine in June 2021. The patient, who was previously fully functional and active, complained of morning stiffness lasting more than an hour, which left her unable to dress herself or perform her daily tasks without assistance. The patient was seen by another physician who prescribed prednisone at 20 mg/d for 3 days. However, she deliberately stopped taking it suddenly due to palpitations. She was then seen by another rheumatologist who prescribed methotrexate at 7.5 mg/wk, just 1 week before her presentation at our clinic. When she was not taking steroids, her inflammatory markers were elevated: CRP was 10.6 mg/L (normal <0.5 mg/L) and ESR was 119 mm/h. The ACPA, RF, and antinuclear antibodies (ANA) tests were negative. No clinical signs of GCA were observed. The chest X-ray was normal. Ultrasound (US) of both shoulders revealed mild right-sided biceps tendon tenosynovitis and subacromial bursitis, along with mild left-sided glenohumeral joint effusions suggestive of polymyalgia rheumatica. Ultrasound of the bilateral hips/thighs revealed a moderate amount of fluid within both hip joints and left-sided gluteus minimus tendinitis with minimal trochanteric bursitis. Therefore, PMR was diagnosed. Prednisone 15 mg/d was restarted, and the dose of methotrexate was increased to 15 mg/wk. A follow up over 1 year demonstrated gradual improvement with standard PMR treatment.

### 2.3. Case 3

A 71-year-old Caucasian female, a former heavy smoker with a history of juvenile idiopathic arthritis diagnosed at age 8 and now in full remission, and dyslipidemia managed with statin therapy, visited the outpatient clinic of LAUMCRH hospital in September 2021 with severe neck and hip pain. Symptoms began 1 day after the second dose of the BNT162b2 COVID-19 mRNA vaccine in March 2021 and have worsened since. The patient reported that the pain was worse in the morning but improved with physical activity. Other symptoms included nighttime awakenings and a limited range of motion. Laboratory workup conducted in June 2021 showed an elevated CRP of 10 mg/dL (normal < 0.5 mg/dL) and an elevated ESR of 94 mm/h. Both RF and ACPA were negative. Upon physical examination, there was severe point tenderness and a decreased range of motion in both shoulders and thighs. Ultrasound scans of both shoulders and hips revealed signs of subacromial and peri-trochanteric bursitis on both sides, supporting the diagnosis of PMR. Therefore, the patient was started on prednisone 10 mg daily (as the patient was reluctant to take high doses of steroids) and Methotrexate 15 mg/wk. A follow up visit in December 2021 showed improved symptoms and increased range of motion, with normalization of both CRP and ESR levels.

Check summary of cases presented in Table 1.

**Table 1:** Three cases of PMR induced by COVID-19 vaccination reported at LAUMCRH Hospital in Lebanon.

Patient number	Age/sex	Type of PMR onset	Type of vaccine	Time between vaccination and symptom onset (d)	CRP & ESR (initially)	Treatment
Case 1	55 yr/M	New onset	BNT162b2 COVID-19 mRNA vaccine	2 wk after 2nd dose	ESR 40 mm/h – CRP 4.2 mg/dL (normal < 0.5 mg/L)	Prednisone 20 mg/d
Case 2	55 yr/F	New onset	BNT162b2 COVID-19 mRNA vaccine	1 d post the 2nd dose	ESR 119 mm/h CRP 10.6 mg/dL (normal < 0.5 mg/L)	Prednisone 15 mg/d and methotrexate 15 mg/wk
Case 3	71 yr/F	New onset	BNT162b2 COVID-19 mRNA vaccine	1 d post the 2nd dose	ESR 94 mm/h CRP 10 mg/dL. (normal < 0.5 mg/L)	Prednisone 10 mg daily and methotrexate 15 mg/wk

## 3. Literature review

Polymyalgia rheumatica is a well-documented condition primarily affecting elderly individuals. It is characterized by pain and stiffness predominantly in the muscles of the neck, shoulders, and hips. Symptoms typically include inflammatory features, worsen in the morning, improve with physical activity, and may include systemic manifestations such as generalized fatigue, weight loss, and low-grade fever. Notably, glucocorticoid therapy often leads to a rapid and significant improvement in symptoms.^[3]^

Although the exact pathogenesis of PMR is still not fully understood, it is believed to involve both genetic and environmental factors. Several infectious agents, including the COVID-19 virus and other viruses, have been identified as potential triggers for PMR.^[2,4–9]^ Additionally, numerous cases reported in the literature document instances of vaccine-induced PMR.^[2,4–9]^

Cases that occurred post non-COVID antiviral vaccines are summarized in Table 2. Several documented cases of PMR and other vasculitides were diagnosed within 3 months after influenza vaccination,^[3,8–12]^ while fewer cases were reported following tetanus and hepatitis vaccinations.^[3,10,13]^ Moreover, multiple other cases occurred after zoster vaccination.^[14,15]^

**Table 2:** Number of individuals reported to have vaccine-induced PMR/GCA.

Vaccines	Individuals with vaccine-induced PMR/GCA	Comments/references
Influenza vaccine	28	○ 10 previously healthy individuals were diagnosed with GCA/PMR < 3 months after vaccination.^[8]^ ○ A 70-yr-old individual experienced a PMR flare-up following vaccination.^[9]^ ○ 3 new onset vaccine-induced PMR/GCA cases.^[2,10,11]^ ○ 4 out of 58 (6.8%) PMR patients were vaccinated before the onset of PMR.^[3]^ ○ 10 out of 358 GCA patients (2.8%) were diagnosed post vaccination.^[12]^
Tetanus vaccine	3	○ A 68-yr-old woman with vaccine-induced PMR and GCA.^[10]^ ○ 2 out of 58 (3.4%) PMR patients received the vaccine before the onset of their disease.^[3]^
Hepatitis B vaccine	4	○ 4 patients were diagnosed with PMR post vaccination.^[13]^
Live attenuated varicella zoster vaccine	39	○ The incidence of new onset GCA was higher in vaccinated individuals (75.2 per 100,000 per year) compared to nonvaccinated individuals (41.6 per 100,000 per year).^[14]^ ○ 55.6% of women with GCA had previously taken the vaccine (39 GCA cases).^[15]^

Check Table 2 which includes the number of individuals reported to have vaccine-induced PMR/GCA.

## 4. COVID vaccines

Regarding the new mRNA vaccines: 2 cases were observed after Ad26.COV2.S, and 31 were observed after mRNA-1273. Forty-two cases were reported after ChAdOx1-S. Four hundred eighty cases were observed after BNT162b2. The cases are summarized in Table 3.

**Table 3:** Number of cases of PMR/GCA reported to be induced by COVID vaccines.

Vaccines	Individuals with vaccine- induced PMR/GCA	Comments/references
Ad26.COV2.S (Janssen)	2	○ 2 cases of PMR were induced by vaccines out of 952,550 individuals vaccinated.^[16]^
mRNA-1273 (Moderna)	31	○ 2 cases of new onset PMR presented within 2 wk after the 2nd dose.^[17,18]^ ○ A 80-yr-old individual presented with a relapse of PMR following the 2nd dose, after being 5 yr in remission.^[19]^ ○ Out of 11,924,026 vaccinated individuals, 22 developed PMR and 6 developed GCA following vaccination.^[16]^
ChAdOx1-S (AstraZeneca)	42	○ 5 cases of PMR/GCA were reported within 1 wk after the 2nd dose.^[20,21,22]^ ○ A 65-yr-old previously healthy female presented with PMR 48 h after the 1st dose.^[23]^ Out of 7,390,842 vaccinated individuals, 27 developed PMR and 9 developed GCA after vaccination.^[16]^
BNT162b2 (Pfizer)	480	○ 3 patients experienced a PMR relapse after being in remission for 1, 2, and 11 yr, respectively.^[17]^ ○ 20 new onset PMR/GCA cases presented within 2 wk after vaccination.^[5,6,17,22,24–32]^ ○ Out of 50,587,266 vaccinated individuals, 128 developed PMR and 39 developed GCA following vaccination.^[16]^ ○ 290 cases of isolated PMR postvaccination.^[33]^

## 5. Discussion

Since December 2020, nearly 4.6 billion doses of the BNT162b2 COVID-19 vaccine have been shipped to 181 countries, including 1.8 billion doses to low- and middle-income countries.^[34]^ In contrast, the ChAdOx1-S, mRNA-1273, and Ad26.COV2.S vaccines have been shipped to a lesser extent, with 2 billion, 641.5 million, and around 5.5 million doses respectively. This could explain the higher frequency of PMR induced by the BNT162b2 vaccine compared to other COVID vaccines.^[35–37]^

According to the nationwide study conducted by Jarrot (2024), when considering the relative risk of both PMR/GCA per adult and the injected dose, we observe a lower incidence of these diseases following COVID-19 vaccination compared to the nonvaccinated population.^[16]^ This finding poses challenges in interpreting pharmacovigilance studies.

Several mechanisms have been proposed to explain the association between infections/vaccines and PMR. These include genetic polymorphisms and age-related immune system dysregulation, which cause an excessively inadequate immune response. Pro-inflammatory markers are released upon exposure to these triggering agents, contributing to the development of PMR.^[2,4–9]^

Three hypotheses have been proposed to explain the potential link between immunization and the development of PMR:

a) Several articles focus on factors that may predispose individuals to PMR following vaccination. For instance, Perez and Maravi (2000) found that cases of influenza vaccination-induced PMR occurred in patients already predisposed with the human leukocyte antigen DRB1*0404 allele.^[38]^ Additionally, Liozon et al (2020) discussed how personal or family history and the predominance of the DRB1*13:01 allele could also play a role in GCA/PMR induced by vaccine adjuvants.^[12]^
b) The second hypothesis is based on the Autoimmune Syndrome Induced by Adjuvants model, which was described in 2011. This entity suggests that certain adjuvants used in vaccine preparation might induce an inappropriate inflammatory/immunogenic reaction in predisposed individuals or those with preexisting autoimmune conditions. This results in symptoms such as generalized malaise, muscle or joint pain, and neurological disturbances.^[3]^ For example, Gherardi (2003) reported that aluminum hydroxide (Al(OH)_3)_ and Th-2 adjuvant, which are present in tetanus, influenza, and hepatitis A and B vaccines, have been associated with the occurrence of PMR.^[37]^ Additionally, squalene, an adjuvant found in the flu vaccine, has been shown to increase the ratio of helper TH17 cells to regulatory T cells, leading to an excessively strong B cell response (Institute of Cellular Medicine, Newcastle University, Newcastle upon Tyne, UK).^[8]^ Although mRNA vaccines do not contain adjuvants, the lipid nanoparticles that deliver the mRNAs into cells, along with the attached polyethylene glycol (PEG) molecules, can produce an immunogenic effect similar to that of adjuvants.^[5]^ This effect could potentially act as a trigger for PMR.
c) The third hypothesis suggests that Toll-like receptors (TLR), specifically TLR7 and TLR9, may contribute to the development of PMR following immunization. These receptors, which are upregulated in PMR patients, could be stimulated by mRNA vaccines. This stimulation may lead to an overactivation of the immune system upon vaccination, subsequently resulting in PMR in genetically susceptible individuals.^[5]^

## 6. Conclusion

As the worldwide spread of SARS-CoV-2 continues to pose significant challenges, vaccination remains a crucial strategy for reducing morbidity and mortality. However, physicians must be aware of all potential vaccine side effects to ensure appropriate diagnosis and provide timely and straightforward management of patients. In all the cases presented above where individuals developed polymyalgia rheumatica post mRNA BNT162b2 vaccine, the clinical presentation closely resembled idiopathic PMR. As is widely known, PMR can be triggered by various viruses, including SARS-CoV-2, and can also be induced by multiple antiviral vaccines.^[2,4–9]^

In this report, we present 3 cases of PMR following vaccination with the BNT162b2 COVID-19 mRNA vaccine. The first patient experienced symptoms 5 days after the second shot, while the remaining 2 patients reported symptoms just 1 day after the second dose of the vaccine. All 3 patients met the European League Against Rheumatism and the American College of Rheumatology 2012 criteria for PMR diagnosis.^[4]^ PMR was diagnosed based on clinical, laboratory, and radiological workup conducted a few days after vaccination with the BNT162b2 COVID-19 mRNA vaccine. Subsequent treatment with systemic prednisone, with or without methotrexate, resulted in an immediate relief of symptoms.^[5,6]^

While the definitive correlation between the COVID-19 vaccine and polymyalgia rheumatica remains uncertain, the cases presented above prompt further investigation, which would ideally include control cohorts of nonvaccinated individuals with PMR/GCA. Another consideration is the possibility of a concomitant asymptomatic infection with the SARS-CoV-2 virus or other viruses during the pandemic waves, which could have triggered PMR without any evidence from testing for these infections. Nevertheless, there appears to be a temporal association between the vaccination and the onset of symptoms. Finally, with a high index of suspicion, these cases can be easily diagnosed and treated properly, preventing any sequelae.

## Author contributions

**Conceptualization:** Laure Irani.

**Formal analysis:** Laure Irani, Mira Bou Karroum, Yara Chehab, Nesrine Abi Saad, Ali Al Dailaty, Roula Husni.

**Investigation:** Laure Irani, Mira Bou Karroum, Yara Chehab, Nesrine Abi Saad, Ali Al Dailaty, Roula Husni.

**Resources:** Mira Bou Karroum, Yara Chehab, Nesrine Abi Saad, Ali Al Dailaty, Roula Husni.

**Supervision:** Laure Irani.

**Validation:** Laure Irani.

**Writing – original draft:** Laure Irani, Mira Bou Karroum, Yara Chehab, Nesrine Abi Saad, Ali Al Dailaty.

**Writing – review & editing:** Laure Irani, Mira Bou Karroum, Yara Chehab, Nesrine Abi Saad, Ali Al Dailaty, Roula Husni.
